# Catalytic Methane Decomposition on In Situ Reduced FeCo Alloy Catalysts Derived from Layered Double Hydroxides

**DOI:** 10.3390/nano14221831

**Published:** 2024-11-15

**Authors:** Dianfeng Cao, Yuwen Li, Chao Lv, Yongtao An, Jiangfeng Song, Mingcan Li, Xin Zhang

**Affiliations:** 1 School of Materials Science and Engineering & Xinjiang Engineering Research Center of Environmental and Functional Materials, Xinjiang University, Urumqi 830046, China; caodf23@163.com; 2 Institute of Materials, China Academy of Engineering Physics, Jiangyou 621908, China; limliayuw@gmail.com (Y.L.); lvchao219@foxmail.com (C.L.); anyt03@163.com (Y.A.); iterchina@163.com (J.S.)

**Keywords:** catalytic decomposition of methane, carbon nanofibers, layered double hydroxides

## Abstract

Catalytic methane decomposition (CMD) reaction is considered a promising process for converting greenhouse gas CH_4_ into hydrogen and high-value-added carbon materials. In this work, a series of Al_2_O_3_-supported FeCo alloy catalysts were successfully prepared in the CMD process. Compared to the pre-reduced catalysts, the in situ reduced FeCo alloy catalysts showed higher methane conversion rates, with the highest reaching 83% at 700 °C, due to the finer active nanoparticle size and greater exposure of active site. Furthermore, the time-on-stream tests demonstrated that the catalytic activity of in situ reduced FeCo alloy catalysts could remain above 92.3% of the highest catalytic activity after 10 h. In addition, TEM analyses of the carbon products from the CMD in situ reduced catalysts revealed the production of carbon nanofibers and nanotubes several microns in length after the reaction. This indicates that the in situ reduced FeCo alloy catalysts more effectively promoted the growth of carbon nanofibers. These results could provide a viable strategy for future methane decomposition development aimed at producing hydrogen and high-value carbon.

## 1. Introduction

Methane is a common chemical raw material employed in the production of hydrogen through methane steam reforming and methane dry reforming. These processes are favored due to methane’s high hydrogen content [1,2,3]. However, traditional methods of hydrogen production often result in impure hydrogen due to the presence of by-products such as CO*x* (CO_2_ or CO) gas. Fortunately, catalytic methane decomposition (CMD) offers a promising alternative for obtaining CO*_x_*-free hydrogen [3,4,5]. Additionally, CMD also generates valuable carbon materials such as carbon nanotubes (CNTs) and carbon nanofibers (CNFs) [6]. These carbon materials have shown great potential for use in various applications, including hydrogen storage materials and electrochemical devices [7].

Since CMD is an endothermic reaction, according to the thermodynamic equilibrium diagram (Figure S1), significant hydrogen production can only be achieved at temperatures of at least 1000 °C without a catalyst [8,9]. In order to reduce the conditions of CMD, it is urgent to use efficient catalysts to reduce the temperature required for catalytic methane decomposition. Currently, the most commonly used efficient catalysts are based on Ni, Co, and Fe elements due to their cost-effectiveness and excellent catalytic performance [10,11]. However, in the CMD process, these active metal nanoparticles tend to sinter or be coated with carbon products, leading to catalyst deactivation [12,13,14]. Therefore, significant research efforts are focused on optimizing catalysts’ performance to enhance their resistance to carbon deposition and to promote the directional selection of highly ordered carbon structures during the CMD reaction [15,16,17]. For example, Qian found that the catalyst with a small particle size reduces the diffusion rate of carbon through the catalyst crystal, resulting in a fast deactivation rate and ultimately a low carbon yield [18]. Additionally, according to Gao’s study, the Ni/Al_2_O_3_ and Co/Al_2_O_3_ catalysts prepared using the aerogel method exhibit strong metal–carrier interaction and possess a high specific surface area. This enhances the catalyst performance compared to those prepared using the impregnation method [19].

In recent years, lots of studies have focused on using simple and well-dispersed layered double hydroxides (LDHs) as precursor for preparing supported catalysts, garnering increasing attention [20]. This interest arises from several advantages of LDHs, including improved controllability, high specific surface area, and uniform dispersion of metal elements [21,22]. In the study of Wang, it was found that the NiFe bimetallic catalyst supported on Al_2_O_3_ prepared using LDH precursors, exhibited a high specific surface area and excellent dispersion of metal particles. This resulted in a higher rate of carbon production in the CMD process [23]. Similarly, in Li’s study, the NiCo/Al_2_O_3_ catalyst prepared through LDH reduction displayed uniform particle size and distribution. It demonstrated high catalytic activity and selective formation of ordered carbon in the CMD reaction [24]. During the CMD reaction, the particle size of the catalyst has a certain correlation with the structure of the carbon material. In the pyrolysis process of LDH, the particle size of the active catalyst can be controlled by adjusting the temperature. Therefore, LDH can be an excellent candidate for preparing CMD catalysts.

In previous literature studies, it was discovered that the composite material, which was prepared using LDH in situ, exhibits better dispersion and has been widely used and proven effective in various reactions, including the oxygen evolution reaction [25,26,27]. Zhang reported that the FeCoNiO*x*(OH)*y* catalyst, prepared in situ using a metal–organic framework material (MOF) as the precursor, not only retains the distinctive structural characteristics of the precursor but also demonstrates a synergistic effect of metal elements, resulting in exceptional catalytic activity in the oxygen evolution reaction (OER) [25]. Gunde Kari discovered that the catalyst prepared using the in situ method has a greater number of active sites and is free from dust particles (particles of dust and other substances in the air), compared to the catalyst prepared by the non-in situ method. When applied to cyclohexanol production, this catalyst achieves an impressive product yield of 98%, with complete conversion of the reactants [26]. Additionally, Kharas found that the catalyst prepared through the in situ reduction method effectively avoids pore plugging poisoning, resulting in improved porosity, stability, and catalytic activity during Fluid catalytic decompositon performance testing [27].

In this work, inspired by the above literature on in situ catalyst preparation, a series of Al_2_O_3_ supporting FeCo alloy catalysts (FeCo/Al_2_O_3_) with different Fe/Co ratios were in situ prepared in the CMD process using FeCoAl-LDHs as precursors. The effect of catalyst preparation methods and element ratio on the CMD performance was studied.

## 2. Experiment

### 2.1. Materials

Iron nitrate nonahydrate (Fe(NO_3_)_3_·9H_2_O), cobalt nitrate hexahydrate (Co(NO_3_)_2_·6H_2_O), aluminum nitrate nonahydrate (Al(NO_3_)_3_·9H_2_O), and urea (CO(NH_2_)_2_) used in the experiment were obtained from Aladdin Chemical Reagent Co, Ltd. (Shanghai, China). The methane gas was supplied by Sichuan Runtai Special Gas Co, Ltd. with a content of 99.999%. All chemical reagents were of analytical purity, without further purification.

### 2.2. Sample Preparation

The FeAl-LDH, CoAl-LDH, and FeCoAl-LDH were prepared using the urea hydrolysis method [28]. Fe(NO_3_)_3_, Co(NO_3_)_2_, and Al(NO_3_)_3_ with molar ratios of 2:0:1, 0:2:1, 1:1:1, 2:1:1, and 3:1:1 were, respectively, mixed with 0.2 mol of urea, dissolved in 150 mL deionized water, transferred into a Teflon-lined stainless-steel autoclave, sealed, and heated under 120 °C for 12 h. After cooling, the reaction precipitates were collected, washed by centrifugal separation until the pH of the supernatant was neutral, then dried at 60 °C for 12 h, and ground into a powder after cooling, and finally, the LDH samples were obtained, named FeAl-LDH, CoAl-LDH, and FeCoAl-LDH-*x* (where *x* = 1–3, refers to the mole ratio of Fe:Co:Al).

For comparison with the FeCoAl-LDH samples, a part of FeAl-LDH, CoAl-LDH, and FeCoAl-LDH-*x* were taken for reduction. FeCoAl-LDH-*x* were reduced under 700 °C for 2 h with a heating rate of 5 °C/min in an atmosphere with 10% H_2_/Ar. After cooling, the samples were collected and named Fe/Al_2_O_3_ and Co/Al_2_O_3_ FeCo/Al_2_O_3_-*x* (where *x* = 1–3, refers to the mole ratio of Fe:Co:Al). (The heating rate of 5 °C/min was selected to avoid the heating furnace temperature exceeding the set temperature when the set temperature was reached. In this work, all heating rates were carried out at 5 °C/min.)

### 2.3. Characterization of Catalysts

Inductively Coupled Plasma (ICP) was carried out using a PE AVIO 200 plasma emission spectrometer. The X-Ray Diffraction (XRD) was carried out using the DX-2700 BH X-ray diffractometer. The Transmission Electron Microscopy (TEM) was carried out using an HT 7800 high-contrast transmission electron microscope. The Brunauer–Emmett–Teller (BET) method was carried out by analyzing the specific surface area using an ASAP 2460 specific surface area and porosity analyzer. Thermogravimetric (TG) was carried out using an STA 449 F3 thermogravimetric analyzer, and the sample was heated to 800 °C at a rate of 5 °C/min. A Raman analysis was performed with a HORIBA AploRA PLUS confocal Raman spectrometer with an excitation wavelength of 532 nm and wave number ranging from 50 to 3400 cm^−1^. The gas phase analysis was performed by a chromatograph system (GC, Agilent 7890 B).

### 2.4. Catalysts’ Activity

In this study, the CMD reaction was carried out using a vertical fixed-bed stainless steel tube reactor, and the sample area in the tube was located in the heating center. In situ catalytic methane decomposition was carried out by 70 mg FeCoAl-LDH-*x* in the CH_4_ with a flow rate of 20 sccm, respectively. A gas chromatography system (GS) was used to monitor the CH_4_ and H_2_ at the reactor outlet. In the CMD reaction, the catalytic performance of the catalyst at different temperatures was tested. After the CH_4_ signal was stable, the reactor was heated to 800 °C at a heating rate of 5 °C/min. In addition, the reactor was heated from room temperature to 700 °C at a heating rate of 5 °C/min and kept warm for a long time to explore the change in methane content with time in the catalytic methane decomposition reaction. Finally, the methane conversion rate was used to evaluate the catalyst activity:

$$\mathrm{Methane}\;\mathrm{conversion}\;\%=\frac{{{\mathrm{CH}}_{4-\mathrm{ave}}-\mathrm{CH}}_{4}}{{\mathrm{CH}}_{4-\mathrm{ave}}}\times 100\%$$

in which CH_4-ave_ and CH_4_ are the integrated areas of the CH_4_ signal in the GS output signal, where CH_4-ave_ is the average integrated area of the stable CH_4_ signal in the unheated state.

## 3. Results and Discussion

### 3.1. Catalyst Characteristics

In order to determine the metal element proportions in the samples, the FeCoAl-LDH-*x* were characterized using ICP analysis, with the results shown in Table S1. The molar ratios of Fe, Co, and Al elements in FeCoAl-LDH-1, -2, -3 were found to be 0.99:1.3:0.97, 2.03:1.02:0.98, and 2.82:1.01:0.99, respectively. These results confirm that the element ratios in the samples are consistent with the intended design values.

**Figure 1:**
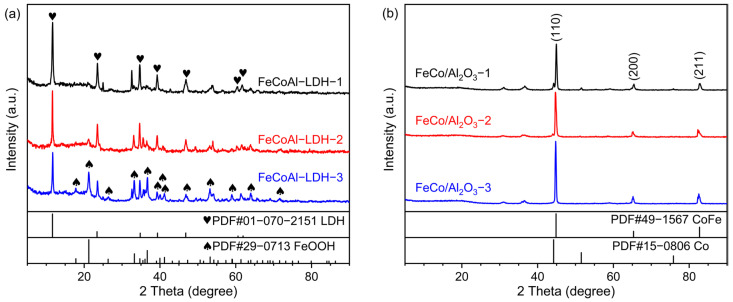
(**a**) XRD patterns of FeCoAl-LDH-*x* and (**b**) XRD patterns of FeCo/Al_2_O_3_-*x*.

The XRD was carried out to determine the crystal phase structure of samples using a 2θ range of 5° to 90° and a step size of 0.02°. As shown in Figure 1a, the diffraction peaks located at 11.65°, 23.51°, 34.67°, 39.28°, 46.82°, 60.33°, and 61.51°, respectively, correspond to the (003), (006), (012), (015), (018), (110) and (113) lattice planes of LDH (PDF#01-070-2151), which proves the successful synthesis of FeCoAl-LDH-*x*. Furthermore, a diffraction peak at 2θ of approximately 20° can be attributed to FeOOH (PDF#29-0713), which is formed during the preparation process due to the high Fe content. As the control group, a part of FeCoAl-LDH-*x* was taken for reduction. After reduction, the XRD patterns of FeCo/Al_2_O_3_-*x* are shown in Figure 1b. The diffraction peaks at about 44.8°, 65.26°, and 82.51° correspond to the (110), (200), and (211) lattice planes of CoFe (PDF#49-1567), respectively, which proves that FeCoAl-LDH-*x* was successfully reduced to form FeCo alloy. At the same time, the XRD patterns of FeCo/Al_2_O_3_-*x* do not reflect the diffraction peaks related to the Al element; this is because, during the reduction process of LDH, Al^3+^ is transformed into amorphous Al_2_O_3_ topological phase transition [29]. To further explore the surface composition and chemical state of catalysts, the X-ray photoelectron spectroscopy (XPS) characterization was carried out. As shown in Figure S2. In Fe 2p and Co 2p spectra of FeCo/Al_2_O_3_-*x*, the peaks detected at 706 eV and 778 eV are assigned to the Fe° and Co°. The ionic states of Fe and Co may be derived from the inevitable exposure to air. In Al 2p spectra of FeCo/Al_2_O_3_-*x*, the peaks detected at 74 eV are assigned to the Al^3+^. Obviously, this further confirms the successful preparation of the FeCo/Al_2_O_3_ catalyst. In order to better understand the structure of the catalyst in the reduced state, a hydrogen temperature programmed reduction (H_2_-TPR) analysis was performed on Fe-CoAl-LDH-*x* material. The result is shown in Figure S3. We found that from room temperature to 800 °C, the minor reduction peak and main reduction peak of FeCoAl-LDH-*x* material appeared at 320 °C and 625 °C, respectively. The minor reduction peak belonged to the reduction of Fe^3+^ and the main reduction peak belonged to the reduction of Fe^2+^ and Co^2+^ in the FeCoAl-LDH-*x* material. In the reduction process of Fe^3+^, it is first reduced to Fe^2+^ and then to Fe°, so there are two reduction peaks during the entire temperature rise process [30]. In addition, due to the different constituent elements, the main reduction peaks of FeCoAl-LDH-1, -2, and -3 appeared at 631 °C, 618 °C, and 613 °C, respectively. This indicates that the reduction peaks of LDH shift to low temperature with the decrease in Co content, indicating that the increase in Co content enhances the interaction between the metal and support in the catalyst.

**Figure 2:**
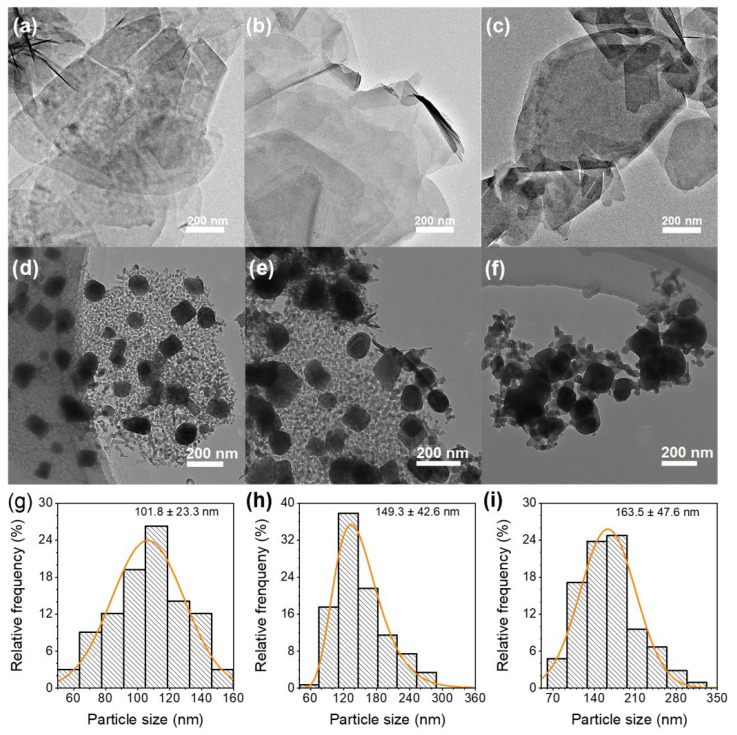
TEM images of (**a**–**c**) FeCoAl-LDH-*x* (*x* = 1–3). (**d**–**f**) TEM images of FeCo/Al_2_O_3_-*x* (*x* = 1–3). (**g**–**i**) Statistical distribution graphs of the particle size of FeCo/Al_2_O_3_-*x* (*x* = 1–3).

To detect the microstructural characteristics of FeCoAl-LDH-*x* and FeCo/Al_2_O_3_-*x*, TEM was carried out. As displayed in Figure 2, the TEM images of FeCoAl-LDH-*x* revealed that the lateral sizes of FeCoAl-LDH-*x* were in the range of hundreds of nanometers, while their thicknesses measured in dozens of nanometers. Similarly, TEM was also conducted for the FeCo/Al_2_O_3_-*x*. As illustrated in Figure 2, the FeCo alloy particles were found to disperse on the Al_2_O_3_ layer after reduction, and an increase in Fe content resulted in particle aggregation. The particle size distribution of the catalytic particles is illustrated in Figure 2d–f. In FeCo/Al_2_O_3_-1, -2, and -3, the predominant size ranges of the catalytic particles were determined to be 100–120 nm, 110–150 nm, and 160–190 nm, respectively. These findings suggest that an increase in Fe content leads to an enlargement in the diameter of the reduced catalytic particles. Furthermore, according to the BET results (shown in Table S2), the specific surface areas of FeCoAl-LDH-1, -2, and -3 are 88.8 m^2^·g^−1^, 64.7 m^2^·g^−1^, and 64.8 m^2^·g^−1^, respectively. And, specific surface areas of FeCo/Al_2_O_3_-1, -2, and -3 are 29.3 m^2^·g^−1^, 27.3 m^2^·g^−1^, and 19.8 m^2^·g^−1^, respectively. Obviously, after reduction, the specific surface areas of FeCo/Al_2_O_3_-*x* significantly decreased, due to the formation of FeCo alloy nanoparticles.

**Figure 3:**
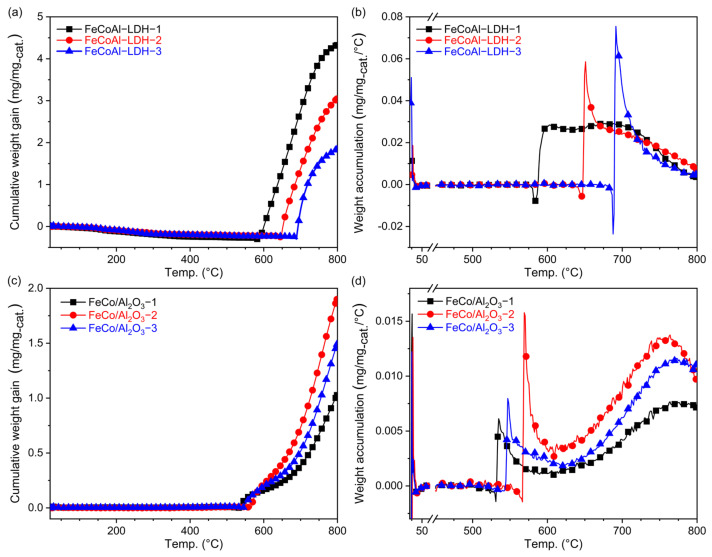
(**a**) TG analysis of the FeCoAl-LDH-*x*, (**b**) the differential curves of TG about FeCoAl-LDH-*x*, (**c**) TG analysis of the FeCo/Al_2_O_3_-*x*, and (**d**) the differential curves of TG about FeCo/Al_2_O_3_-*x*.

A TG analysis was conducted to monitor the change in mass with temperature during the in situ CMD reaction. The heating process started from 20 °C and reached 800 °C at a heating rate of 5 °C, with a mixed flow of 20 sccm CH_4_ and 20 sccm Ar. As shown in Figure 3a, the mass of the reactants exhibited a slight decrease before reaching 600 °C, which corresponds to the reduction phase of FeCoAl-LDH-*x*. After reaching 600 °C, the mass gradually started to increase, indicating carbon accumulation. The masses of FeCoAl-LDH-1, -2, and -3 began to increase at about 588 °C, 648 °C, and 696 °C, respectively. The variation in temperature at which the catalyst mass increases is attributed to the difficulty of reducing Fe. The differing Fe content in the catalysts means that those with higher Fe concentrations require a higher temperature range to be fully reduced before catalyzing the CMD reaction and accumulating carbon materials, leading to an increase in weight. With increasing temperature, the mass of the carbon product continued to accumulate until the end of the heating process. The normalized data revealed that the carbon accumulation for FeCoAl-LDH-1, -2, and -3 reached 4.33 mg/mg_-cat_, 3.05 mg/mg_-cat_, and 1.84 mg/mg_-cat_, respectively. These results indicate that an increase in Fe content leads to a decrease in carbon accumulation, as higher Fe loadings reduce the catalyst’s surface area [31].

To understand the relationship between carbon accumulation rate and temperature, the differential curves of carbon accumulation amount and the temperature are shown in Figure 3b. As the Fe content increased, the temperature at which the carbon accumulation rate started to increase and the maximum value reached also increased. Specifically, the carbon accumulation rates of FeCoAl-LDH-1, -2, and -3 reached their maximum values at 601 °C, 650 °C, and 690 °C, respectively, with values of 0.03 mg/(mg_-cat_·°C), 0.06 mg/(mg_-cat_·°C), and 0.08 mg/(mg_-cat_·°C). As the temperature continued to rise, the carbon accumulation rate passed the maximum and gradually declined. FeCoAl-LDH-3 exhibited the fastest decay, followed by FeCo-LDH-2, while FeCoAl-LDH-1 maintained the longest time. In a previous study on Fe-based catalysts, single-metal Fe-based catalysts required temperatures of above 700 °C to demonstrate good methane decomposition performance [21]. Our above findings suggest that the Co element can reduce the activation energy of the CMD reaction and sustain carbon accumulation.

For comparison, the catalytic effect of the pre-reduced FeCo/Al_2_O_3_-*x* catalysts on the CMD reaction was also analyzed using TG, and the corresponding curves are shown in Figure 3c. The FeCo/Al_2_O_3_-*x* catalysts started accumulating carbon at around 550 °C. As the temperature increased, the carbon accumulation continued to increase until the heating process ended. Among them, the carbon accumulation of FeCo/Al_2_O_3_-1, -2, and -3 reached 1.03 mg/mg_-cat_, 1.90 mg/mg_-cat_ and 1.49 mg/mg_-cat_, respectively, which is lower than that of FeCoAl-LDH-*x*. Among them, the FeCo/Al_2_O_3_-2 catalyst showed the largest carbon material accumulation sequence, nearly twice that of FeCo/Al_2_O_3_-3 and FeCo/Al_2_O_3_-1. To analyze the relationship between carbon accumulation rate and temperature, the differential curves are presented in Figure 3d. The carbon accumulation rate of FeCo/Al_2_O_3_-*x* started to increase after 520 °C, and with increasing temperature, the carbon accumulation rate rapidly reached a maximum value and then decreased. Near the end of the heating process, it reached a second maximum value. The maximum accumulation rates of FeCo/Al_2_O_3_-*x* are all smaller than that of FeCoAl-LDH-*x*. In summary, under the same CH_4_ flow rate and temperature program, the FeCo/Al_2_O_3_-*x* catalysts exhibited lower carbon accumulation and carbon accumulation rates compared to the FeCoAl-LDH-*x* catalysts.

### 3.2. Performance Test of Catalysts

**Figure 4:**
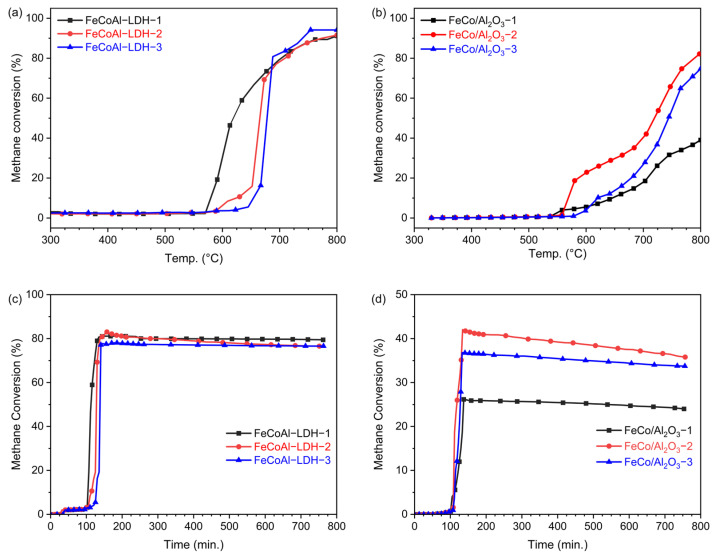
The relationship between methane conversion and temperature in the TPSR test at 5 °C/min to 800 °C for (**a**) FeCoAl-LDH-*x* and (**b**) FeCo/Al_2_O_3_-*x*, and the relationship between methane conversion and temperature in the TOS test at 5 °C/min to 700 °C for insulation of (**c**) FeCoAl-LDH-*x* and (**d**) FeCo/Al_2_O_3_-*x*.

To investigate how catalyst activity changes with temperature, a temperature-programmed methane decomposition reaction was conducted in a fixed bed, and Figure 4a illustrates the methane conversion rate of FeCoAl-LDH-*x*. As the temperature increases, the methane conversion rate gradually rises to its maximum value. Under the same mass, the maximum methane conversion rates for FeCoAl-LDH-1, -2, and -3 are 92.5%, 91.6%, and 94.2%, respectively. Figure 4b displays the methane conversion rate of FeCo/Al_2_O_3_-*x*. The temperature at which the methane conversion curve in the reaction of the fixed bed begins to rise is essentially the same as that at which the carbon deposition curve starts to rise in the TG test. The difference between the starting temperature of carbon deposition in FeCoAl-LDH-*x* observed in the TG test and the rising temperature of methane conversion in the fixed bed reaction is attributed to the fact that methane is initially used for catalyst reduction in the fixed bed test. Therefore, the starting temperature of methane conversion change is lower than that of carbon deposition in the TG reaction, which differs from the TG test. Initially, the weight reduction change in the catalyst coincides with the methane conversion rate. However, after the complete reduction of FeCoAl-LDH-*x*, the methane conversion rate of FeCoAl-LDH-*x* is higher than that of FeCo/Al_2_O_3_-*x* without in situ reduction. Comparing the methane conversion curves of FeCo/Al_2_O_3_-*x*, it can be observed that the methane conversion rate decreases with an increase in Fe content. On the other hand, in the methane conversion curve of FeCoAl-LDH-*x* prepared through in situ reduction, the methane conversion rate increases. This indicates that the in situ reduction method results in better metal dispersion and more active sites on the catalyst, thereby avoiding a decrease in methane conversion caused by a reduction in specific surface area due to an increase in Fe content. In addition, to explore the effect of Fe and Co forming alloys on the CMD performance, the temperature-programmed CMD activity of FeAl-LDH, CoAl-LDH, Fe/Al_2_O_3_, and Co/Al_2_O_3_ catalysts was tested. As shown in Figure S4, in situ reduction consistently exhibits better performance than the pre-reduced catalysts, which is consistent with the results of the alloy catalyst. Under the same mass and test conditions, the maximum methane conversion rates of FeAl-LDH, Fe/Al_2_O_3_, CoAl-LDH, and Co/Al_2_O_3_ are 8.1%, 7.3%, 86.4%, and 69.1%, respectively. After the formation of the FeCo alloy, the highest methane conversion rate can reach 94.2%. The improved activity of alloy catalysts relative to single metal catalysts is attributed to the interaction between the metals and the alloying effect.

Additionally, time-on-stream tests of FeCoAl-LDH-*x* and FeCo/Al_2_O_3_-*x* were carried out. The time-on-stream tests were conducted with a heating rate of 5 °C min^−1^ and held at 700 °C. As shown in Figure 4c, during the heating stage, the methane conversion rate of FeCoAl-LDH-*x* changes by very little. However, once the optimal catalytic temperature is reached, the methane conversion rate quickly reaches its maximum value. The maximum methane conversion rates of FeCoAl-LDH-1, -2, and -3 are 81.1%. 83.0%, and 78.1%. After reaching 700 °C and reacting for 650 min, the methane conversion rates of FeCoAl-LDH-1, -2, and -3 can maintain maximum values of 97.9%, 92.3%, and 98.1%, respectively. As a comparison, the time-on-stream tests of FeCo/Al_2_O_3_-*x* were conducted and the results are shown in Figure 4d. The maximum methane conversion rates of FeCo/Al_2_O_3_-1, -2, and -3 are 26.1%, 42.0%, and 36.7%. After reaching 700 °C and reacting for 650 min, the methane conversion rates of FeCo/Al_2_O_3_-1, -2, and -3 can maintain maximum values of 92.0%, 80.2%, and 97.5%, respectively. Compared to the FeCo/Al_2_O_3_-*x* catalyst, the activity and stability of the FeCoAl-LDH-x catalyst are superior, which may be attributed to the finer active nanoparticle and formation of ordered carbon. Additionally, we have summarized the activity and stability of several Fe-based catalysts in the CMD reaction and compared them with our findings in this paper, as shown in Table S3. Under the same conditions, the FeCo alloy catalysts prepared by in situ reduction in this work showed comparable performance in terms of reaction temperature and methane conversion.

### 3.3. Spent Catalyst Characteristics

**Figure 5:**
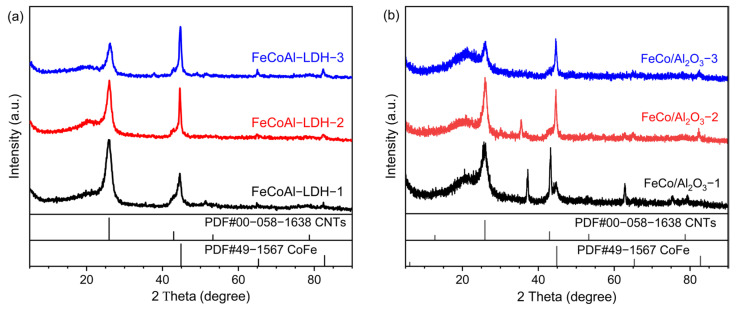
XRD patterns of methane decomposition reaction products catalyzed by (**a**) FeCoAl-LDH-*x* and (**b**) FeCo/Al_2_O_3_-*x*.

To confirm the synthesis of FeCo alloy in the in situ reduced FeCoAl-LDH-*x* catalysts, XRD patterns were obtained and are presented in Figure 5. In the 2θ range of 5° to 90°, the diffraction peak at approximately 26.57° corresponds to the diffraction peak of CNTs (PDF#00-05801638), while the diffraction peaks at around 44.60°, 65.03°, and 82.35° correspond to the (110), (200), and (211) crystal planes of CoFe (PDF#49-1567). Based on these findings, it can be concluded that FeCo alloy was successfully synthesized through the in situ reduction of FeCoAl-LDH-*x*. Furthermore, with an increase in Fe content, the intensity of the CoFe diffraction peaks becomes stronger, while the intensity of the C diffraction peak becomes weaker. This phenomenon suggests that during the in situ reduction CMD reaction, the reduction of FeCoAl-LDH-*x* and the synthesis of FeCo alloy proceed simultaneously. Since the TG analyses were conducted under the same temperature program, the reaction time for the in situ reduction of catalytic particles with higher Fe content is longer, while the CMD reaction time is shorter.

**Figure 6:**
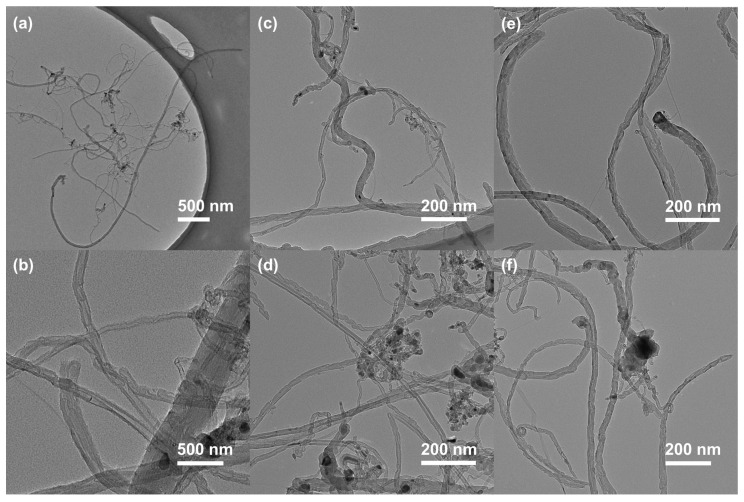
TEM images of carbon products created by (**a**,**b**) FeCoAl-LDH-1, (**c**,**d**) FeCoAl-LDH-2, (**e**,**f**) and FeCoAl-LDH-3.

A TEM analysis was performed to examine the morphology of the carbon products obtained from the FeCoAl-LDH-*x* in situ CMD reaction, and the results are shown in Figure 6. Firstly, the carbon nanomaterials produced by FeCoAl-LDH-*x* include carbon nanofibers (CNFs) with a diameter of approximately 45 nm and a length of several micrometers (Figure 6a,c,e), as well as bamboo-shaped carbon nanotubes (CNTs) with a diameter of about 20 nm (Figure 6b,d,f). The TEM results indicate that the majority of carbon nanotubes possess catalytic particles at their tips, suggesting that the most carbon nanomaterials are synthesized via a top-growth mechanism. Furthermore, the catalytic particles at the top of the carbon product were counted, and the average size of the FeCo alloy particles in FeCoAl-LDH-*x* after in situ reduction was approximately 72.2 nm. This size is significantly smaller compared to that observed in FeCo/Al_2_O_3_-*x* catalysts. Moreover, the initial LDH structure was not observed in the TEM image; instead, FeCo alloy nanocrystals were identified. This finding confirms that the FeCo alloy catalysts were successfully in situ synthesized in the CMD process.

**Figure 7:**
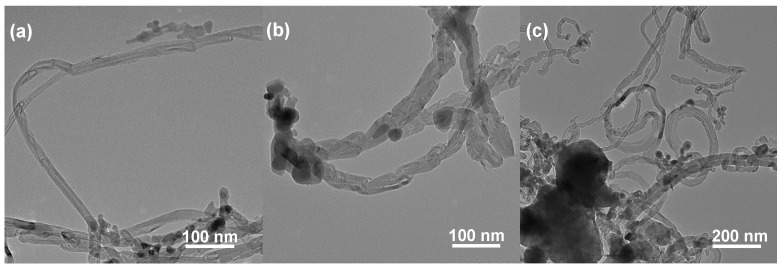
TEM images of TG products created by (**a**) FeCo/Al_2_O_3_-1, (**b**) FeCo/Al_2_O_3_-2, and (**c**) FeCo/Al_2_O_3_-3.

The TEM images of the FeCo/Al_2_O_3_-*x* products obtained after the TG reaction are presented in Figure 7. The products of the FeCo/Al_2_O_3_-*x* TG reaction consist of bamboo-shaped carbon nanotubes (CNTs) with a diameter of approximately 20 nm (Figure 7a) and carbon nanofibers (CNFs) with a diameter of about 30 nm (Figure 7b). In comparison to FeCoAl-LDH-*x*, the FeCo/Al_2_O_3_-*x* particles were found to be more prominently encapsulated by carbon deposits. This explains why FeCo/Al_2_O_3_-*x* exhibits less carbon accumulation during the TG reaction than FeCoAl-LDH-*x*. Furthermore, the catalytic particles at the top of the carbon product from FeCo/Al_2_O_3_-*x* were counted, and it was determined that the average size of the FeCo alloy particles was approximately 110.12 nm. This value is larger than that observed in FeCoAl-LDH-*x*. This difference may indicate that the particle size after the in situ reduction of FeCoAl-LDH-*x* is more suitable for the growth of CNFs with lengths on the order of several nanometers.

**Figure 8:**
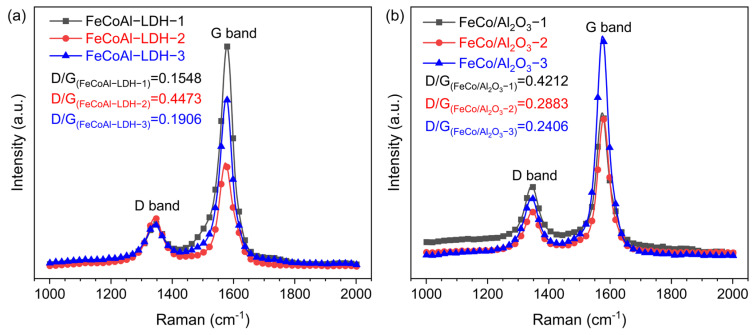
Raman spectra of (**a**) spent FeCoAl-LDHs-*x* (*x* = 1–3) and (**b**) FeCo/Al_2_O_3_-*x* (*x* = 1–3) catalysts after TG.

To obtain information on the carbon products, the Raman spectra were carried out. As shown in Figure 8, the characteristic D and G bands of carbon material were clearly observed at 1343 cm^−1^ and 1579 cm^−1^, respectively, for all catalysts. The D band typically arises from structural disruptions in graphite layers or the presence of amorphous carbon [32]. On the other hand, the G band is typically associated with C-C tensile vibrations in sp^2^ hybridized planes [32,33]. The presence of a prominent G band indicates the growth of graphitized or ordered carbon nanostructures. Notably, in Figure 8, the intensity of the G band for all catalysts is significantly higher than that of the D band, suggesting the existence of ordered or crystalline carbon [33]. Among them, the D/G of carbon materials produced by each catalyst is, respectively, D/G_(FeCoAl−LDH−1)_ = 0.1584, D/G_(FeCoAl−LDH−2)_ = 0.4473, D/G_(FeCoAl−LDH−3)_ = 0.1906, D/G_(FeCo/Al2O3-1)_ = 0.4212, D/G_(FeCo/Al2O3-3)_ = 0.2883, and D/G_(FeCo/Al2O3-3)_ = 0.2408. The D/G ratio for the LDH catalysts is approximately half that of the FeCo alloy catalyst, indicating that the LDH catalyst is more favorable for the growth of crystalline carbon. Furthermore, the TEM image shown in Figure 6 reveals the absence of amorphous carbon in all samples, which explains the high intensity of the G band. This may be attributed to the ordered carbon structure resulting from the bundle structure. Fluctuations in absorption peaks at 1180 cm^−1^ and 1510 cm^−1^ are typically associated with sp^3^ carbon materials, which could be related to defects in the CNFs and CNTs.

## 4. Conclusions

In this work, a series of FeCo alloy catalysts with different Fe/Co ratios were successfully synthesized using LDH as a precursor by in situ reduction. The in situ prepared FeCoAl-LDH-*x* catalyst demonstrates a superior carbon accumulation ratio compared to the pre-reduced FeCo/Al_2_O_3_-*x* catalyst. TEM observations reveal that the carbon products derived from the FeCoAl-LDH-*x* catalyst consist of numerous CNFs and CNTs with lengths of several micrometers. Moreover, in the Raman spectroscopy analysis of carbon products, the carbon products from the in situ prepared FeCoAl-LDH-*x* catalyst exhibits a lower D/G ratio, which signifies a preferential accumulation of crystalline carbon. The results of the CMD reaction performance test show that the methane conversion of FeCoAl-LDH-2 can reach 83% at 700 °C. The time online test proved that the catalytic activity of the FeCoAl-LDH-*x* catalyst remained above 92.3% of the highest catalytic activity after 10 h. Compared with the Fe-based catalysts in former reported studies, the in situ reduced catalysts in this paper showed comparable CMD performance under similar test conditions. These findings provide an effective strategy for the preparation of highly active, highly stable CMD catalysts that can produce high-value carbon materials and hydrogen.

## Data Availability

Data are contained within the article and Supplementary Materials.

## Supplementary Materials

The following supporting information can be downloaded at https://www.mdpi.com/article/10.3390/nano14221831/s1, Figure S1: Equilibrium conversion of methane at different temperatures; Table S1: ICP tests’ raw data and processing of FeCoAl-LDH-*x*; Table S2: BET surface area (m^2^/g) test results of FeCo/Al_2_O_3_-*x*. Figure S2. XPS spectra of (a) survey, (b) Fe 2p, (c) Co 2p, (d) Al 2p. Figure S3. H_2_-TPR profiles of the FeCoAl-LDH-x. Figure S4. The relationship between methane conversion and temperature in the TPSR test at 5 °C/min to 800 °C for (a) FeAl-LDH and Fe/Al_2_O_3_; (b) CoAl-LDH and Co/Al_2_O_3_. Table S3: Comparison of performance of Fe-based catalysts. (References [34,35,36,37,38,39,40,41,42,43,44] have been cited in Supplementary Materials).
