# Supplementary material for: Catalytic Methane Decomposition on In Situ Reduced FeCo Alloy Catalysts Derived from Layered Double Hydroxides
Source: Nanomaterials (Basel). 2024 Nov 15;14(22):1831. doi: 10.3390/nano14221831 (PMC11597559; doi:10.3390/nano14221831)
Supplement: Supplementary file 1 [file nanomaterials-14-01831-s001.zip › nanomaterials-3257683-supplementary.pdf]

Catalytic Methane Decomposition on In Situ Reduced FeCo Alloy Catalysts Derived from Layered Double Hydroxides

Dianfeng Cao [1,2], Yuwen Li [2], Chao Lv [2], Yongtao An [2], Jiangfeng Song [2], Mingcan Li [1,*] and Xin Zhang [2,*]

[1] School of Materials Science and Engineering & Xinjiang Engineering Research Center of Environmental and Functional Materials, Xinjiang University, Urumqi 830046, China; caodf23@163.com
[2] Institute of Materials, China Academy of Engineering Physics, Jiangyou 621908, China; limliayuw@gmail.com (Y.L.); lvchao219@foxmail.com (C.L.); anyt03@163.com (Y.A.); iterchina@163.com (J.S.)
* Correspondence: limingcan@xju.edu.cn (M.L.); zhangxin23@caep.cn (X.Z.)

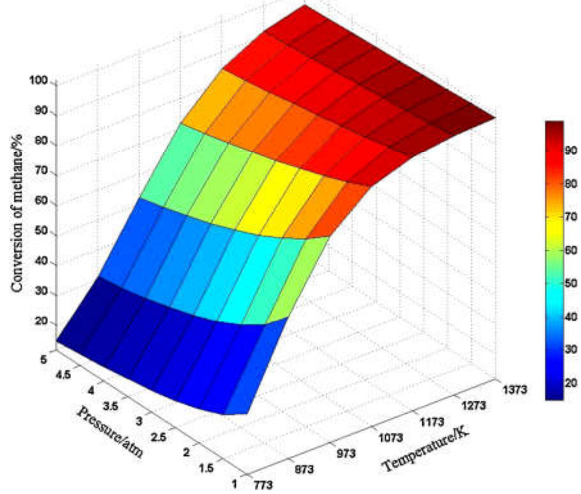

Figure S1. Equilibrium conversion of methane at different temperatures [1].

1.1 FeCo/Al_2O_3 Catalyst characteristic

Table S1. ICP tests raw data and processing of FeCoAl-LDH-*x*.

Sample	Measured element	Instrument reading (mg/kg)	n (mol) (×10^{-5})	n_{Fe}:n_{Co}:n_{Al}
FeCoAl-LDH-1	Fe	201320.0	7.3	1.0:1.0:1.0
	Co	221198.0	7.6	
	Al	95084.0	7.2	
FeCoAl-LDH-2	Fe	328342.6	11.9	2.0:1.0:1.0
	Co	174399.5	6.0	
	Al	76127.2	5.7	
FeCoAl-LDH-3	Fe	359713.8	13.0	2.8:1.0:1.0
	Co	136683.1	4.7	
	Al	60857.2	4.6	

Table S2. BET surface area (m^2/g) test results of $FeCo/Al_2O_3$-*x*.

Sample	BET surface area (m^2 g^{-1})	Single-point pore volume at $P/P_0 = 0.99$(cm^3 g^{-1})	BET average pore diameter (nm)
FeCoAl-LDH-1	88.8	0.3	14.9
FeCoAl-LDH-2	64.7	0.2	10.6
FeCoAl-LDH-3	64.8	0.2	11.7
$FeCo/Al_2O_3$-1	29.8	0.2	20.9
$FeCo/Al_2O_3$-2	27.3	0.1	21.3
$FeCo/Al_2O_3$-3	19.8	0.1	28.0

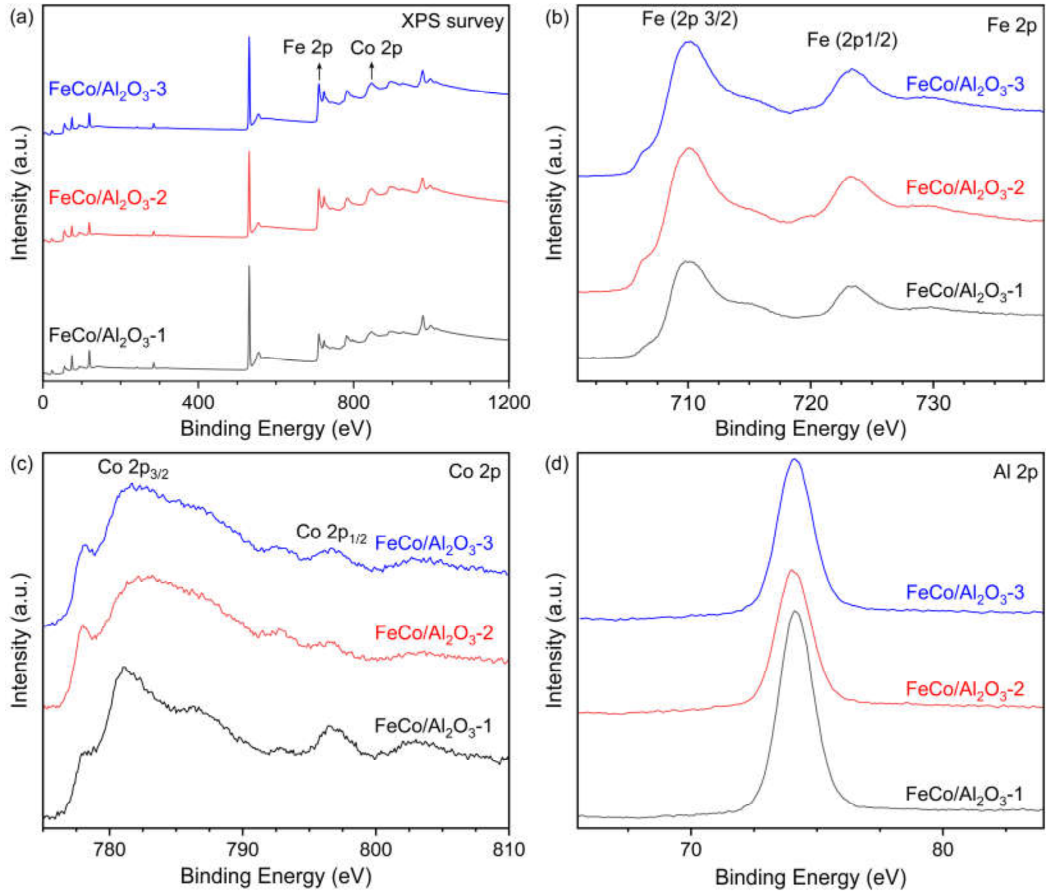

Figure S2 XPS spectra of (a) survey, (b) Fe 2p, (c) Co 2p, (d) Al 2p.

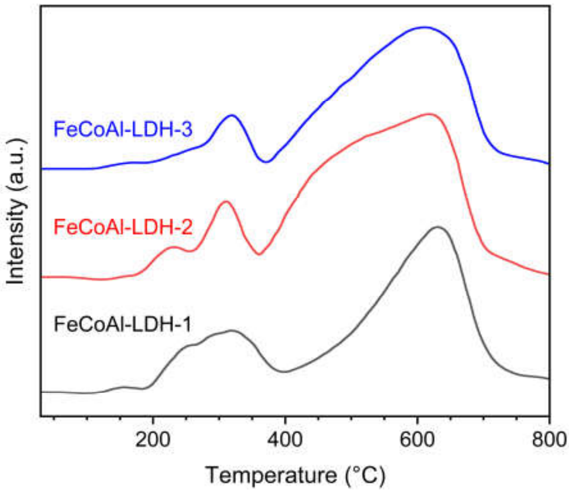

Figure S3 H_2-TPR profiles of the FeCoAl-LDH-*x*.

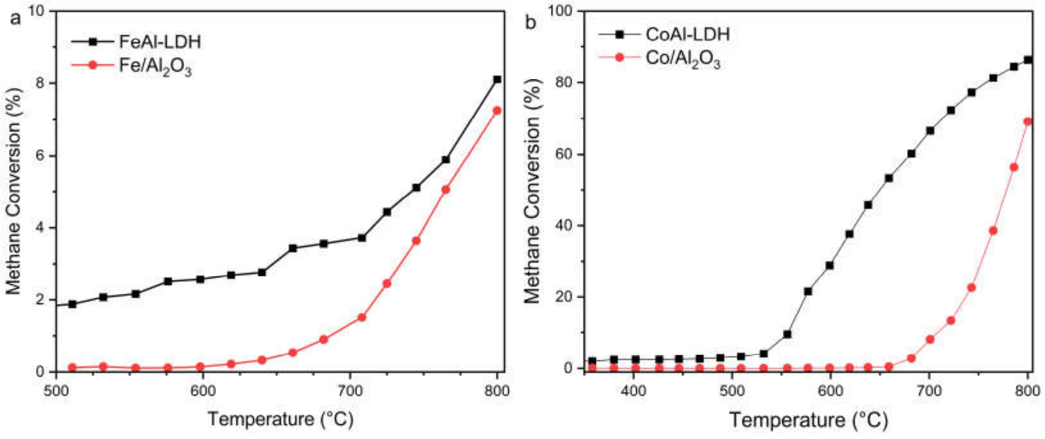

Figure S4 The relationship between methane conversion and temperature in TPSR test at 5 °C/min to 800 °C (a) FeAl-LDH and Fe/Al_2O_3; (b) CoAl-LDH and Co/Al_2O_3.

Table S3. Comparison of catalytic performance of Fe-based.

Catalysts	Weight/mg	reactor	Reduction condition		Stability and activity maintaine		Intial CH_4 conversion (%)	Final CH_4 conversion (%)	H_2 yield $mol_{H2}/g_{cat}/h$
			T_1(°C)	T_2(h）	T_1(°C)	T_2(h）			
59.5%Fe/15%SiO_2[2]	15	FLBR	-	-	680	9	-	-	0.76
50%Fe/Al_2O_3[3]	-	VFR	580	5	625	23	4	0	0.19
50Fe/6%Co/Al_2O_3[3]	-	VFR	580	5	625	40	8	0	0.22
60%Fe/15%SiO_2[4]	15	VFR	700	-	700	9	-	-	0.5
Unsupported Fe[5]	1000	-	600	1.5	900	6	-	-	0.18

Unsupported bulk Fe[6]	250	FBR	800	1	900	2	-	-	0.27
Iron ores (Wate) [7]	1600	FLBR	850	3.5	850	9	62	50	0.36
Tiergaore,52.6%Fe[8]	600	FBR	900	1	800	3	30	32	-
Fe-Al[9]	500	-	500	4	800	6	-	95.5	-
Fe/CNT [10]	10000	FBR	500	3	700	40	85	72	-
Fe-Ni-Ce/γ-Al [11]	100	-	700	1	750	5	93	96	-
Fe-Ni/ γ -Al_2O_3 [11]	100	-	700	1	750	5	97	28	-
12%Fe/Al_2O_3[12]	20000	FBR	500	3	700	2	84	55	-
FeCoAl-LDH-2 (This work)	70	FBR	-	-	700	10	83	76.6	-
FeCo/Al_2O_3-2 (This work)	70	FBR	700	2	700	10	42	33.7	-

References:

1. Y. Li, D. Li, G. Wang, Methane decomposition to COx-free hydrogen and nano-carbon material on group 8 – 10 base metal catalysts: a review. Catal. Today, (2011) 162 1-48. [http://doi.org/doi.org/10.1016/j.cattod.2010.12.042]

2. M.A. Ermakova, D.Y. Ermakov, A.L. Chuvilin, G.G. Kuvshinov, Decomposition of methane over iron catalysts at the range of moderate temperatures: the influence of structure of the catalytic systems and the reaction conditions on the yield of carbon and morphology of carbon filaments. (2001) 201 183-197.

3. L.B. Avdeeva, T.V. Reshetenko, Z.R. Ismagilov, V.A. Likholobov, Iron-containing catalysts of methane decomposition: accumulation of filamentous carbon. (2002) 228 53-63.

4. M. Ermakova, D.Y. Ermakov, Ni/SiO_2 and Fe/SiO_2 catalysts for production of hydrogen and filamentous carbon via methane decomposition. (2002) 77 225-235.

5. M. Pudukudy, Z. Yaakob, N. Dahani, M.S. Takriff, N.S.M. Hassan, Production of CO*x* free hydrogen and nanocarbon via methane decomposition over unsupported porous nickel and iron catalysts. (2017) 28 1579-1594.

6. A.E. Awadallah, A.A. Aboul-Enein, U.F. Kandil, M.R. Taha, Facile and large-scale synthesis of high quality few-layered graphene nano-platelets via methane decomposition over unsupported iron family catalysts. (2017) 191 75-85.

7. L. Zhou, L.R. Enakonda, S. Li, D. Gary, P. Del-Gallo, Christina Mennemann, J.M. Basset, Iron ore catalysts for methane decomposition to make CO*x* free hydrogen and carbon nano material. (2018) 87 54-63.

8. J.A. Silva, J.B.O. Santos, D. Torres, J.L. Pinilla, I. Suelves, Natural Fe-based catalysts for the production of hydrogen and carbon nanomaterials via methane decomposition. (2021) 46 35137-

35148.
9. E. Abdel-Fattah, M.A. Alotaibi, A. Alharthi, Thermo-catalytic methane decomposition over unsupported Fe–Al and Co–Al catalysts for hydrogen and carbon nanostructures production. (2024) 64 685-694.
10. M. Yang, J. Baeyens, S. Li, Z. Li, H. Zhang, Catalytic methane decomposition on CNT-supported Fe-catalysts. (2024) 365 121592.
11. N. Makayeva, G. Yergaziyeva, S. Soloviev, E. Kutelia, L. Nadaria, O. Tsurtsumia, B. Zhuginis, M. Annisova, M. Mambetova, K. Dossumov, Effects of cerium oxide on the activity of Fe-Ni/Al2O3 catalyst in the decomposition of methane. (2024) 161 112047.
12. M. Yang, J. Baeyens, S. Li, H. Zhang, Hydrogen and carbon produced by fluidized bed catalytic methane decomposition. (2024) 204 67-80.
